# Effects of waste-based concentrates from broiler farm on physico-chemical qualities and blood profile of landrace pigs

**DOI:** 10.5455/javar.2023.j712

**Published:** 2023-11-04

**Authors:** Tirta Ariana, Djoko Kisworo, Bulkaini Bulkaini, Sumerta Miwada, Dewi Ayu Warmadewi, Rahayu Tanama Putri

**Affiliations:** 1 Faculty of Animal Science, University of Udayana, Badung, Indonesia; 2 Faculty of Animal Science, University of Mataram, Mataram, Indonesia

**Keywords:** Blood profile, landrace pigs, meat, physico-chemical

## Abstract

**Objective:**

The study aimed to determine the effect of giving concentrate protein from closed-house (CP-CH) broiler farm waste in rations on the meat quality (physical-chemical) and blood profile of landrace crossbreed pigs.

**Materials and Methods:**

The study used a completely randomized design (CRD) with 3 treatments and 4 replications with 12 finisher-phase landrace pigs with an average body weight of 63.42 ± 2.39 kg. The treatments were: A (control): use of 0% CP-CH + 24% CP-152 concentrate; B: 12% CP-CH + 12% CP-152 concentrate; and C: 24% CP-CH + 0% CP-152 concentrate. The research parameters were meat quality (physical-chemical meat) and blood lipid profile [total cholesterol, high-density lipoprotein (HDL), low-density lipoprotein (LDL), and triglycerides (TGs)].

**Results:**

The results showed that treatment group B obtained physical meat quality (pH, drip loss, cooking loss, and water holding capacity) and meat chemical quality (moisture, protein, fat, and ash content) that were almost the same as the control (*p >* 0.05). By administering 24% CP-CH + 0% CP-152 (C) concentrate, there was a significant decrease in the physical and chemical quality of crossbreed landrace pigs (*p* < 0.05). Administration of CP-CH at 12%–24% did not affect total cholesterol, HDL, LDL, or blood TGs in landrace-crossbreed pigs.

**Conclusion:**

Giving 12% CP-CH (B) obtained meat quality (physical and chemical) almost the same as the control group. Treatment group C significantly reduced the physical and chemical quality of the meat. Administration of CP-CH at 12%–24% did not affect the blood profile of landrace crosses.

## Introduction

Good physical and chemical qualities of meat are the main requirements for its acceptance by consumers. Handling pigs before slaughter, such as nutrition, climate or temperature, fatigue, and excessive movement, can affect post-mortem muscle metabolism, meat quality, and blood profile [1,^11^,19]. In raising pigs, feed costs are still the highest (70%–80%), and the problem causes breeders to always look for alternative feed sources that are less expensive but do not reduce their nutritional value [12]. Several research reports on the use of agricultural and industrial agricultural waste can be used as feed ingredients or supplements in animal feed to increase production, carcass quality, and meat quality [6,^10^,^14^,^19^,37]. Utilization of other agricultural wastes, such as residues of fermentation of broccoli stems and leaves and supplementation of plant essential oils, can affect growth, the quality of the pork finishing phase, and the biochemical characteristics of serum [22,23]. In the production process of broiler chicken farms using the closed-house system, by-products are found in the form of culled chickens and litter scattered by leftover feed [2,4]. However, the by-product has the potential to be used as a protein source in the preparation of non-ruminant livestock rations (specifically pigs), after which it is referred to as by-product-based protein concentrates for closed-house broiler farms or “CP-CH” [2].

Pigs are very prospective as meat producers and continue to experience an increase in population, meat production, and slaughter [17]. The good performance of pigs has great potential as a meat producer. This can be seen in the response of farmers, who are pretty good at increasing the efficiency of the production and reproduction of pigs [16]. Pigs that experience stress are chemically in a state of muscle glycogen deficiency, increased blood glucose levels, and weight loss caused by the loss of body fluids [drip loss (DL)] [20]. Factors such as nutritional management, breed, and general condition of pigs before slaughter can influence carcass composition, meat quality, and blood characteristics of pigs, such as blood lipid profiles [24,^25^,^27^,33].

Blood tests are performed as a screening procedure to evaluate the general health of pigs. A blood lipid profile can be determined by measuring total cholesterol, triglycerides (TGs), and high-density lipoprotein (HDL). A lipid profile is a measure of the risk of cardiovascular disease [17]. Hematological values can be used as indicators of the response of pigs to the environment, and the feed given [35] conveys that blood is one of the parameters of the immune system that determines the health status of animals because blood has components that function very well in the physiological regulation of the body. It is also said that the feed given has become a choice, so it is necessary to know its effect on livestock’s mean total erythrocytes and hemoglobin levels. Variations in physiological numbers in blood parameters such as hematological profiles and clinical symptoms are significant as an indication of the tolerance of pigs to feed and the environment. The effect of feed management on blood chemistry profiles, such as substituting concentrates with mulberry leaf flour, can significantly affect blood glucose, cholesterol, and HDL levels [36]. Research using cassava pellet supplementation of 0%, 20%, and 40% on the basal feed did not affect growth performance or blood profile [26].

The above conditions inspired us to study the quality (physical and chemical), meat, and blood fat profiles of landrace pigs fed protein concentrates from broiler farm waste using the closed-house system (CP-CH).

## Materials and Methods

### Animal ethics agreement

This study was approved by the Ethics Committee of the Faculty of Veterinary Medicine at Udayana University, Bali, Indonesia (approval number: B/272/UN14.2.9/PT.01.04/2022). Landrace pigs with an average body weight of 63.42 ± 2.39 kg used in this study were handled professionally (in accordance with the management of pig rearing). During the experiment, landrace pigs were placed in colony cages with a size of 3.0 × 3.0 m^2^ and four landrace pigs per pen. During the study, the landrace pigs were given food and drink according to their treatment. At the end of the study, the experimental animals were slaughtered according to the requirements for slaughtering pigs at the slaughterhouse to obtain research samples.

### Blood sample

Blood samples were taken through the jugular vein, as much as 2–3 ml, using a venoject tube without anticoagulants. Total cholesterol, TGs, and HDL levels were measured in pig blood serum using a reagent kit and photometrically with a semi-automatic biochemistry analyzer photometer 5010 v5+. Total cholesterol levels were determined using the cholesterol oxidase-peroxidase aminoantypirin method [8]. The method uses the principles of enzymatic oxidation and hydrolysis. Ten microliters of serum was reacted with 1,000 μl of cholesterol reagent and then incubated at 37°C for 5 min. There are two kinds of cholesterol reagents used, namely enzyme reagents and standard reagents. The determination of TGs levels was conducted using the glycerol peroxidase phosphate acid method. This method uses the same principle as the determination of total cholesterol, namely, oxidation and enzymatic hydrolysis; the difference is the reagent used. Precipitation was carried out to determine HDL by adding 1,000 µl of HDL cholesterol (HDL-CHOL) reagent and 500 µl of sample serum to a test tube, by homogenizing and incubating for 10 min at 15°C–25°C, and then by centrifuging for 10 min at 4,000 rpm. Then, the supernatant was separated from the precipitate. After centrifugation, HDL in the supernatant is determined using the HDL-CHOL kit reagent.

### Meat quality test (physical-chemical)

Meat samples for meat quality (physical and chemical) testing were taken from the longissimus dorsi muscle. Thirty-six meat samples represented 3 treatments and 12 replications. The meat consists of physical properties such as water-holding capacity (WHC), cooking loss (CL), and pH value. Testing of the pH value was carried out using the Ockerman method [38], which involved briefly dissolving 5 gm of meat sample with 45 ml of distilled water for 2 min, and the pH meter electrode was dipped into the solution until a stable number was obtained. WHC was measured by the Hamm method [32], in which a 0.3 gm meat sample was placed between two filter papers and weighed at 35 kg for 5 min. The covered area and the wet area of the meat sample were measured using a planimeter. The difference between the two areas was calculated to determine the wet area (stained area − meat area). The formula for calculating the moisture content of meat was: MgH_2_O = [(wet area (cm^2^) × (0.0948) − 1] − 0.8. The WHC of meat was calculated by the formula: WHC = total moisture content − [(mg H_2_O) × (sample weight) −1] × 100%. The value of meat CL was calculated by looking at the difference between the weight of the meat before and after cooking, dividing by the weight of the meat before cooking, and multiplying by 100% [39].

### Research design

The research design used was a one-way, completely randomized design (CRD) with three treatments and four replications. The study used 12 finisher-phase pigs with an average body weight of 63.42 ± 2.39 kg. Reared for 70 days and slaughtered with an average slaughter weight of 103.92 ± 8.51 kg. The treatment given to the research is as follows:

- Ration with 24% CP-152 concentrate and 0% CP-CH (Control).
- Ration with 12% CP-152 concentrate and 12% CP-CH.
- Ration with 0% CP-152 concentrate and 24% CP-CH.

### Concentrate protein from closed-house

Concentrates from the wastage or by-products from closed-house system chicken farming (CP-CH) is a protein source consisting of dead broiler chicken flour and litter flour mixed with spilled feed. The rejected and dead chickens were chopped and baked in the oven at 70°C for 2 × 24 h. After drying, it is followed by grinding to get meat flour. Litter flour is obtained from litter collection in an area of 10 cm around the feed area (480 pieces per cage closed house), and then mixed thoroughly and dried under the sun until air-dried. Then, it is milled until it becomes litter flour. To increase the nutritional value and safety of the feed as a ration constituent, it is further fermented with EM-4. CP-CH flour consists of two parts of litter flour and one part of rejected chicken meat flour. The nutritional content of CP-CH is presented in Table 1 [2]. Commercial concentrate produced by limited company Charoen Pokphand Indonesia, with code CP-152 [21], is a protein source for mixed pig rations from the grower phase to the finisher phase. The nutritional content of CP.152 and CP-CH concentrates is shown in Tables 1–3.

### Research parameters

The research parameters consisted of the physical quality of meat: pH, DL, CL, and WHC; meat chemical quality: protein content, fat content, ash content, and meat water content; and blood lipid profile: measurement of total cholesterol, TGs, HDL, and low-density lipoprotein (LDL).

**Table 1.** Concentrated nutritional content of CP-152 and CP-CH.

No	Nutrient	CP.152 (%) [21]	CP-CH (%) [2]
1	Water content	12.0 (max)	11.3
2	Ash	20.0 (max)	10.4
3	Crude protein	37.0 (min)	39.7
4	Crude fat	3.0 (min)	4.8
5	Coarse fibre	8.0 (max)	8.4
6	Calcium	3.0–5.0	15.2
7	Phosphor	1.2–2.0	1.2
8	Gross energy (kcal/gm)	3,654	5,110

**Table 2.** The composition of the research rations.

Ingredients	Treatment (%)		
	A (Control)	B	C
Concentrate CP.152	24	12	0
CP-CH	0	12	24
Polar	35	35	35
Corn	40	40	40
Salt	1	1	1
Total	100	100	100

**Table 3.** Nutritional content of pork ration according to treatment.

No.	Analysis	Unit	Treatment^a^		
			A	B	C
1	Dry material	%	86.71	87.73	85.59
2	Water	%	13.29	12.27	14.41
3	Ash	%	12.31	15.32	11.30
4	Organic compound	%	87.69	84.68	88.70
5	Crude protein	%	22.86	20.78	19.41
6	Coarse fibre	%	4.01	5.17	7.15
7	Crude fat	%	4.60	5.52	5.97
8	TDN	%	84.32	71.61	67.76
9	BETN	%	32.93	41.93	45.76
10	Gross energy	kcal/gm	3.73	3.15	3.33

a Results of nutritional analysis of research rations at the Laboratory of Feed and Nutrition, Faculty of Animal Science, Udayana University (2022).

### Data analysis

The research data were analyzed using analysis of variance and Duncan’s multiple range test [18]. The analysis procedure uses SPSS version 22.0.

## Results

### Physical quality of meat

The effect of CP-CH administration on the physical quality of landrace pork is shown in Table 4. The use of 12% commercial concentrate CP-152 + 12% CP-CH (treatment B) found that the pH of meat was almost the same as the pH of meat in treatment group A (control) (*p* > 0.05). The same thing happened to other meat physical qualities, such as the parameters of DL, CL, and WHC, which found the same results as the control treatment group (*p >* 0.05). The use of commercial CP-152 concentrate 0% + 24% CP-CH (C treatment) can significantly affect the physical quality of meat, such as meat pH (5.68), DL (18.13%), and CL (11.14%), which are significantly (*p* < 0.05) higher than the control treatment. Administration of 0% commercial concentrate CP-152 and 24% CP-CH obtained WHC values of 32.98% ± 3.08%, or 7.67%, significantly smaller than the control treatment group (*p* < 0.05).

### Meat chemical quality

In Table 4, the chemical quality parameters of meat for moisture, protein, fat, and ash content in treatment group B obtained results that were almost the same as those in treatment group A (control) (*p >* 0.05). In treatment group C, it was seen that all parameters of the chemical quality of the meat were significantly different (*p* < 0.05) from the control treatment. Moisture content in treatment C: 70.23% ± 0.54% or 4.44% significantly less than the control treatment (*p* < 0.05); protein content in treatment C: 22.32% ± 0.58% or 6%, 81% less than the control treatment (*p* < 0.05); and fat content: 4.45% ± 0.35% or 25.35% higher than the control treatment (*p* < 0.05). Administration of 0% commercial concentrate CP-152 and 24% CP-CH found an ash content of 2.45% ± 0.92%, which was not statistically significantly different from the ash content of the control treatment group (*p >* 0.05).

### Blood lipid profile of landrace pig crosses

The results of Duncan’s analysis of variance and the follow-up tests of the blood lipid profile (total cholesterol, HDL, TGs, and LDL) of landrace pig crosses are shown in Table 5. Administration of CP-CH at 12%–24% in treatment groups B and C resulted in total cholesterol that was almost the same as the control (*p >* 0.05). In treatment group C, the HDL value was 63.92% ± 15.23%, or 1.08% less than the control (*p >* 0.05), and the LDL value in treatment group C was 17.03% ± 18.21%, or 4.41% greater than the control (*p >* 0.05). TGs in treatment group C (73.85 ± 11.14 mg/dl) were significantly (*p* < 0.05) 7.7% lower than the control group and significantly (*p* < 0.05) lower by 11.18% from treatment group B (*p* < 0.05).

**Table 4.** Physical-chemical quality of crossbreed landrace pork given “CP-CH.”

Physical						
Treatment	pH		DL (%)		CL (%)	WHC (%)
A	5.50 ± 0.01^b^		10.81 ± 0.42^b^		36.01 ± 1.07^a^	35.51 ± 2.46^a^
B	5.51 ± 0.01^b^		10.79 ± 0.98^b^		36.36 ± 0.91 ^a^	35.37 ± 2.46^a^
C	5.68 ± 0.01^a^		12.77 ± 2.07^a^		40.02 ± 3.62 ^b^	32.98 ± 3.0^8^b
SEM	0.03		0.14		0.15	0.56
**Chemical**						
**Treatment**		**Water (%)**		**Protein (%)**	**Fat (%)**	**Ash (%)**
A		73.35 ± 0.71^a^		23.84 ±0.67^a^	3.55 ± 0.55^b^	2.91 ± 0.76^a^
B		72.75 ± 0.34^a^		23.22 ± 0.89^a^	3.28 ± 0.79^b^	2.82 ± 0.88^a^
C		70.23 ± 0.54^b^		22.32 ± 0.58^b^	4.45 ± 0.35^a^	2.45 ± 0.92^a^
SEM		0.49		0.17	0.15	0.07

Different superscripts in the same column show a significant difference (*p* < 0.05). SEM: Standard error of the treatment means.

**Table 5.** Blood lipid profile of landrace pig crosses given “KP-CH.”

Treatment	Cholesterol (mg/dl)	HDL (mg/dl)	LDL (mg/dl)	TGs (mg/dl)
A	93.41 ± 5.11**^a^**	93.41 ± 5.11**^a^**	16.31 ± 3.31**^a^**	79.52 ± 12.23**^a^**
B	94.91 ± 7.39**^a^**	94.91 ± 7.39**^a^**	16.86 ± 4.21**^a^**	82.11 ± 10.12**^a^**
C	93.11 ± 8.11**^a^**	93.11 ± 8.11**^a^**	17.03 ± 5.32**^a^**	73.85 ± 11.14**^b^**
SEM	3.11	3.11	1.82	2.33

Different Superscript values in the same column indicate a significant difference (*p* < 0.05). SEM: Standard error of the treatment means.

## Discussion

### Physical-chemical properties of meat

The pH value obtained in treatment group C (5.58 ± 0.01) was significantly higher (*p* < 0.05) than the control treatment group (*p* < 0.05). This was due to the crude protein content of diet C (19.41%), or 17.8% lower than crude protein diet A (Table 3). The gross energy content in CP-CH is 5,110 kcal/gm, which is greater than the gross energy content of CP-152 commercial concentrate (3,654 kcal/gm). The energy content of the feed consumed by livestock will affect the muscle glycogen content [19]. Changes in meat pH are closely related to muscle glycogen reserves when cattle are slaughtered. Meat pH is influenced by intrinsic factors such as glycogen reserves, variations among livestock, and species [11,19]. The pH value of the research results (pH 4.5) was lower than the pH of meat stored at 39°C, which was 6.23 ± 0.01 [13,27]. The optimal pH of meat in general is 5.4–5.5, depending on the ambient temperature [19]. Pork quality is influenced by livestock factors before being slaughtered, such as sex, slaughter weight, nutrition, temperature at slaughter, and transportation [11,^13^,19]. Another opinion states that differences in sex and differences in slaughter weight do not affect pH [27]. This study used the landrace finisher phase with the same initial body weight (63.42 ± 2.39 kg), the same cage environment (temperature, cage density), and the only difference in ration treatment.

### Blood lipid profile

The blood lipid profile was determined by the content of total cholesterol, HDL, LDL, and TGs [7]. Total cholesterol obtained between treatments (A, B, and C) was not significantly different, and these values were within the normal range and considered safe and healthy for consumers. Cholesterol content below 200 mg/dl [28] is declared safe. Cholesterol in landrace blood in this study was influenced by the factor of the feed given with good and almost the same nutritional content. Eighty percent of cholesterol is produced in the body, and 20% comes from feed [29,34]. High and low levels of cholesterol in the blood are affected by several factors, such as type of feed, livestock, genetics, sex, and age of livestock [30]. Increased cholesterol in the blood, apart from affecting health risks, also affects the quality of the meat produced from these livestock. Increased blood cholesterol can increase the cholesterol content of meat [7]. Information about supplementation with 0%, 20%, and 40% cassava pellets into pig rations did not significantly affect growth performance or blood profile [15,26].

The blood HDL and LDL levels of landrace pigs in this study were not significantly different between treatments (*p >* 0.05) because the research uses the same material (breed, initial weight, cage environment). Giving CP-CH 12%–24% in the ration did not cause significant changes in total cholesterol, HDL, or LDL cholesterol in landrace pigs (*p >* 0.05). These results were higher than those reported by Najib et al. [7]. The addition of 10% rejected milk to the CP-550 commercial ration of male pigs after weaning resulted in cholesterol levels of 70.22 mg/dl, HDL of 30.44 mg/dl, and LDL of 23.64 mg/dl, but the TGs level was higher, namely, 92.33 mg/dl. This difference is due to the age of the livestock and the rations given [7,^10^,30]. The high crude fiber content of the ration causes the feed flow rate to increase in the digestive tract, and cholesterol in the ration will exit through intestinal peristalsis. While bile salts will be reabsorbed into the blood to be recirculated as cholesterol [7,31]. One indicator that can be used to assess meat quality is the measurement of HDL and LDL values. Meat that has high HDL (good cholesterol) values and low LDL (bad cholesterol) values [32] is considered good and safe for consumption. HDL and LDL are lipoproteins that function to circulate cholesterol in the blood due to which their concentration in the blood is greatly affected by the amount of cholesterol synthesized. HDL belongs to the group of good cholesterol. HDL levels can reduce the risk of atherosclerosis by transporting cholesterol from peripheral tissues to the liver and reducing excess cholesterol [7,^9^,10].

TGs treatment group C (73.85 ± 11.14 mg/dl) was significantly (*p* < 0.05) lower than control and treatment B. It was caused by the high content (7.15%) of crude fiber in ration C (Table 3). Feeding with a higher fiber content will cause a decrease in blood lipid profiles [10], especially cholesterol, LDL, and TGs levels. The results of this study are in accordance with the opinion of Bulkaini et al. [10], who found that adding fermented pineapple skin to the ration of male Bali cattle could cause their cholesterol levels to be lower than without pineapple skin fermentation or control. The decrease in cholesterol and TGs levels was caused by an increase in crude fiber contained in the research ration. The increase in the crude fiber content of the ration causes the flow rate of the ration to increase so that the cholesterol in the ration will exit through the intestinal tract, while bile salts will be reabsorbed into the blood to be recirculated as cholesterol [10,31].

The weakness of this study is that it has not examined the content of a number of saturated and unsaturated fatty acids, so further research is still needed in an effort to discuss more comprehensively the relationship between cholesterol levels and levels of saturated and unsaturated fatty acids.

The crude protein content in the C treatment ration (19.41%) was lower than the control ration crude protein, but the crude fiber content (7.15%) and crude fat content (5.97%) (Table 3) were higher than the control ration. These conditions significantly affect the decrease in meat quality, especially in the parameters pH, WHC, DL, and CL. Rations with relatively low nutrient content and high crude fiber and crude fat content tend to decrease the WHC of meat (WHC) and increase CLs and DL [5,19]. CL is directly proportional to DLs but inversely proportional to WHC. High meat WHC will decrease CL and DL values [19]. The physical quality of the meat in this study was in accordance with this opinion because the ration content in treatment C, which used 0% commercial concentrate, slightly reduced the nutritional content of the ration.

The chemical quality of the meat is supported by the parameters of water content, protein, fat, and meat ash, as well as the microbial content of the meat [20]. The protein content of the meat obtained in this study was 70.23%–73.35%. The water content of the meat was still within normal limits because the water content of fresh meat is 68%–80% [19]. The water content of male Bali cattle fed 20% fermented pineapple skin ranged from 72.81% to 73.01% [10]. The water content in treatment C was 70.23%, significantly lower than the control. It was because the C ration contains crude protein at 19.41% (lower than control) and crude fiber at 7.15% (higher than control) (Table 3). The high nutritional content of the ration, especially the balance of protein and energy in the ration, can cause an increase in the ability of meat protein to bind water for the better, and this condition can lead to an increase in meat WHC and changes in other meat quality parameters [19,25]. The water content of the meat is strongly affected by the WHC and the fat content of the meat [11]. Meat fat content is inversely proportional to meat protein and water content. The ability of meat protein to bind water can cause a decrease in the water content of the meat, increasing dream loss and CL [10,^11^,19]. It was stated that the chemical quality of pork with a slaughter weight of 110 kg obtained a meat moisture content of 72%, a protein content of 23.4%, and a meat fat content of 2.0% [13]. The slaughter weight in this study was 103.92 ± 8.51 kg unsex, and the chemical quality of the meat was obtained as shown in Table 4. The sex of the pig has no effect on protein, ash, dry matter, WHC, or meat texture [24].

## Conclusion

The use of 12% CP-CH in ration (B) did not affect the quality (physical and chemical) of crossbreed landrace pigs. The use of CP-CH up to 24% in the ration causes a decrease in the physical quality (pH, DL, CL, and WHC) and chemical quality (moisture, protein, and fat) of crossbreed landrace pigs. The use of CP-CH at 12%–24% had no significant effect (*p >* 0.05) on the blood lipid profile (total cholesterol, HDL, LDL, and TGs) of landrace pigs.

## List of abbreviations

CP-CH, concentrate protein closed house; HDL, high-density lipoprotein; LDL, low-density lipoprotein; HDL-CHOL, high-density lipoprotein-cholesterol; WHC, water holding capacity; CL, cooking loss; DL, drip loss; TGs, triglycerides; PT, college code; SPSS, Statistical Package for the Social Sciences.
